# Trauma exposure and stress‐related disorders in African‐American women with diabetes mellitus

**DOI:** 10.1002/edm2.111

**Published:** 2020-01-14

**Authors:** H. Drew Dixon, Vasiliki Michopoulos, Rachel L. Gluck, Hadrian Mendoza, Adam P. Munoz, Joseph G. Wilson, Abigail Powers, Ann C. Schwartz, Guillermo E. Umpierrez, Charles F. Gillespie

**Affiliations:** ^1^ Department of Psychiatry and Behavioral Sciences Emory University School of Medicine Atlanta Georgia; ^2^ Yerkes National Primate Research Center Atlanta Georgia; ^3^ Division of Endocrinology Department of Medicine Emory University School of Medicine Atlanta Georgia

**Keywords:** diabetes, glycaemic control, MDD, PTSD, trauma exposure

## Abstract

**Objective:**

The purpose of the study was to assess demographic features, rates of trauma exposure, prevalence of post‐traumatic stress and depressive symptoms in a group of urban, low‐income, African‐American women with type 1 or type 2 diabetes mellitus.

**Research Design and Methods:**

We conducted a survey of (n = 290) low‐income, African‐American women seeking care in the diabetes clinic of an urban hospital and collected data on the demographic characteristics, childhood and nonchildhood abuse trauma exposure, and the severity of post‐traumatic stress and depressive symptoms using the Post‐traumatic Stress Disorder (PTSD) Symptom Scale (PSS) and the Beck Depression Inventory (BDI). In a subset of women with type 2 diabetes (n = 96), we assessed haemoglobin A1c to examine the relationship between psychopathology and glycaemic control.

**Results:**

Of the overall sample, 61.7% reported exposure to trauma in their lifetime, and 30.4% and 29.3% had current PTSD and MDD, respectively. Exposure to both childhood and nonchildhood abuse trauma was associated with an increased PTSD and depressive symptom severity (*P*'s < .05). PTSD diagnosis, but not depression, was associated with increased haemoglobin A1c (*P* = .002).

**Conclusions:**

These data document high levels of trauma exposure, PTSD and depressive symptoms in diabetic African‐American women treated in a specialty clinic of an urban hospital setting. Furthermore, these data indicate that the presence of PTSD is negatively associated with glycaemic control.

## Graphical abstract

Levels of trauma exposure, post‐traumatic stress disorder (PTSD) and depressive symptoms are high in diabetic African‐American women and adversely impact glycaemic control.

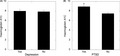

## 1 INTRODUCTION

In the United States, diabetes affects over 25 million Americans with greater prevalence observed in African American (18.7%) relative to Caucasian (10.2%) individuals.^1^ Cardiovascular disease (CVD) and associated risk factors including hyperglycaemia, dyslipidaemia and hypertension occur at a greater rate in African‐American individuals relative to other ethnic groups. Socioeconomic status (SES) may play an important role in this health disparity.^2^ Low SES is strongly associated with increased exposure to traumatic events as well as elevated rates of post‐traumatic stress disorder (PTSD) and major depressive disorder (MDD).^3^, ^4^ Among civilians, economically disadvantaged African Americans living within urban environments experience particularly high levels of trauma and are at increased risk for adverse mental health outcomes.^3^, ^4^, ^5^

Posttraumatic stress disorder is a severely debilitating, stress‐related psychiatric illness associated with exposure to a traumatic experience. Clinically, PTSD is a heterogeneous disorder whose presentation is comprised of variable combinations of re‐experiencing, avoidance, negative mood and hyperarousal symptoms.^6^ In the general population, the lifetime prevalence of PTSD has been estimated to be 5%‐10%^7^ with higher rates of PTSD being observed among combat veterans (30.9% lifetime prevalence)^8^ and individuals living in areas of high violence (17.1% lifetime prevalence).^3^, ^4^, ^5^, ^9^ Like PTSD, MDD is also commonly observed in populations exposed to trauma^4^ and is often comorbid with PTSD.^7^ MDD is characterized clinically by the presence of “depressed” mood or anhedonia in conjunction with at least three to four additional symptoms of disturbed sleep, altered appetite, inappropriate feelings of guilt, impaired concentration, psychomotor changes or suicidal thoughts.^6^

The presence of PTSD and/or MDD has adverse effects on physical health. Patients with comorbid PTSD and MDD have more negative perceptions of their individual health^10^, ^11^ and incur higher healthcare costs than individuals with either MDD or PTSD alone.^12^ In addition, comorbidity of PTSD and MDD is predictive of increased risk for metabolic disorders^13^, ^14^, ^15^ and CVD.^14^, ^16^, ^17^ Further, comorbidity of PTSD and MDD also is associated with severity of hypertension^18^ and metabolic syndrome.^14^ Finally, recently reported prospective data indicate that the presence of PTSD is associated with increased risk for developing type 2 diabetes in traumatized women.^19^ However, the mechanisms responsible for this elevation in cardiometabolic risk in those with PTSD and MDD remain unclear.

To date, the prevalence of trauma exposure, PTSD and MDD specifically in diabetic individuals remains unclear. In the present study, we describe the demographic characteristics, rates of childhood and nonchildhood abuse trauma exposure and the extent of PTSD and MDD symptoms in a group of low‐income, African‐American women with type 1 or type 2 diabetes mellitus recruited from a specialty diabetes clinic of an urban hospital. Additionally, in a subgroup of women with type 2 diabetes, we assessed the impact of MDD and PTSD diagnoses on haemoglobin A1c concentrations, a diabetes‐related biomarker. We hypothesized that MDD and PTSD would be associated with poor glycaemic control and higher levels of haemoglobin A1c.

## 2 METHODS

### 2.1 Overall sample, recruitment and procedure

Study participants (n = 290) were approached by study staff in the waiting room of the diabetes clinic of Grady Memorial Hospital in Atlanta, GA, from 2013 to 2015.^4^ Recruitment was not limited to specific criteria, and study staff approached any individual in the clinic. Participants were informed that the study represented a confidential survey examining their trauma exposure during childhood and adulthood and diabetes control. Eligibility requirements for the study included the ability to give informed consent, having a phone number by which they could be contacted, and the ability to speak and comprehend English. Additionally, participants had to be between the ages of 18 and 65. Exclusion criteria included mental retardation, active psychosis, hospitalization for mental health reasons within the past month, and acute impairment from drugs or alcohol to the degree that they could not provide informed consent. Written and verbal informed consent was obtained for all agreeing participants. Those who consented completed a number of self‐report measures (described below) that took between one and two hours to complete, depending on the participant's self‐report of their trauma history and psychiatric symptoms. At the conclusion of the survey, participants were compensated $15 each for their participation. The Emory Institutional Review Board and the Research Oversight Committee of Grady Memorial Hospital, Atlanta, GA approved all study procedures, which conform with the United States policy for the Protection of Human Subjects and the Declaration of Helsinki.

#### 2.1.1 Measures

The demographics form assesses subject age, self‐identified race and ethnicity, relationship status, education, monthly income, employment and disability status, legal history, frequency of religious practice, brief psychiatric history and diabetes treatment status.^4^

The Traumatic Events Inventory (TEI) is a 14‐item screen for lifetime history of traumatic events.^20^ For each event, the TEI separately assesses experiencing and witnessing of events. Additionally, the TEI also assesses frequency of trauma exposure within each type of trauma type. The total number of types of trauma exposure experienced and witnessed was used in the current data analysis because prior work has shown reliably that types of trauma exposure are associated with a number of measures of adaptive functioning and trauma‐related functioning.^4^ The Childhood Trauma Questionnaire‐Short Form (CTQ‐SF)^21^ is a 28‐item retrospective self‐report questionnaire used to assess childhood abuse. It captures 5 dimensions of abuse including physical abuse, emotional abuse, sexual abuse, physical neglect and emotional neglect. The response items are scored from 1 to 5 (never true, rarely true, sometimes true, often true, very often true) and are structured to capture frequency of childhood maltreatment experiences (higher scores indicate higher levels of childhood maltreatment exposure). Based on the CTQ and the TEI, categorical child and nonchildhood abuse trauma exposure was defined as none, one type and ≥ two types of trauma exposure.

The Modified PTSD Symptom Scale (MPSS) is a psychometrically valid 17‐item self‐report scale assessing PTSD symptoms over the previous 2 weeks.^22^ The MPSS frequency items (“−:not at all” to “3:≥5 times a week”) were summed to obtain a continuous measure of symptom severity ranging from 0 to 51, and to compute a categorical diagnosis of current PTSD.^22^ The MPSS had a standardized alpha coefficient of 0.91 in the current sample. The Beck Depression Inventory (BDI) was administered to assess for current symptoms of depression using 21 items to compute both a continuous measure of depression symptoms and a categorical diagnosis of current depression.^23^ The BDI had a standardized alpha coefficient of .93 in the current sample.

### 2.2 Subsample recruitment and procedures

Study participants with type 2 diabetes were offered the opportunity to participate further in our study by providing a fasted blood sample and undergoing a structured clinical interview to assess the effects of PTSD and MDD on the biology underlying diabetes (n = 96). Type 2 diabetes status was determined for each participant by electronic medical records. We recently reported data from this subset study showing that higher concentrations of c‐reactive protein (CRP) were associated with PTSD and MDD in 55 women who had CRP data available.^24^

#### 2.2.1 Structured clinical interview

Participants were administered a semi‐structured clinical interview which included the assessment of aspects of daily functioning and psychological functioning and diagnoses. For the present study analyses, only diagnoses of PTSD and MDD are included. All study interviews underwent rigorous training by licensed clinical psychologists on the study team on all assessment measures included in the battery and engaged in weekly supervision with one of the licensed psychologists throughout data collection. The Clinician‐Administered PTSD Scale (CAPS) is a well‐validated and reliable diagnostic instrument used to measure the categorical presence of current PTSD^25^ based on criteria from the Diagnostic and Statistical Manual (DSM). CAPS for DSM‐IV‐TR (CAPS‐IV) and DSM‐V (CAPS‐V) was both used, as we switched measures mid‐way through the study upon the release of the CAPS‐V. Thirty‐two per cent (n = 25) of the sample received the CAPS‐IV, and 67.5% (n = 52) received the CAPS‐V. We could determine whether participants who received the CAPS‐V would meet DSM‐IV‐TR diagnostic criteria for PTSD because all DSM‐IV diagnostic criteria were still asked. PTSD symptom severity was calculated as the sum of PTSD symptoms on CAPS. Interrater reliability (IRR) has been examined in this sample and demonstrated good IRR for detecting presence of current PTSD (*k* = 0.83).^26^ The Mini‐International Neuropsychiatric Interview (MINI) is a reliable and well‐validated measure that was administered to assess the presence of a current MDD diagnosis based on DSM‐IV‐TR criteria.^27^

#### 2.2.2 Sample collection and processing

On the morning of the clinical interview (09:00), a fasted blood sample was collected at the Clinical Interactions Network within the Atlanta Clinical and Translational Science Institute for assessment of haemoglobin A1c. Haemoglobin A1c concentrations were assayed by Associated Regional and University Pathologists (ARUP) using quantitative high performance liquid chromatography/boronate affinity chromatography from Trinity Biotech (http://www.trinitybiotech.com).

### 2.3 Statistical analyses

Descriptive statistics and frequencies were used to summarize demographic characteristics, psychiatric history, legal history, frequency and types of trauma exposure, severity of PTSD and depressive symptoms, and prevalence of PTSD and MDD in study participants. We performed an ANOVA to determine whether increasing exposure to different types of childhood and nonchildhood abuse trauma influences PTSD and depressive symptoms, using the MPSS and BDI total scores as dependent variables and categorical child and nonchildhood abuse trauma exposure as independent variables constructed from the CTQ and TEI, respectively. Finally, we performed an ANOVA to determine whether MDD and PTSD diagnoses (determined by the MINI and CAPS measures during structured clinical interview) were associated with haemoglobin A1c in a subgroup of women with type 2 diabetes. All data are presented as mean ± standard error of the mean (SEM). All analyses were conducted with spss 22.0 software package, and a *P* ≤ .05 was considered statistically significant.

## 3 RESULTS

### 3.1 Demographic, legal, physical, diabetic treatment status and general psychiatric characteristics

Table 1 summarizes the demographic characteristics of the study population. The mean age of study participants was 51.5 ± 9.6 years. With respect to educational status, 35.6% (n = 103) listed 12th grade as highest level of education and 26.6% (n = 77) completed some college or technical school. The majority of the participants (n = 212, 73.4%) were unemployed at the time of the screen, and 38.4% (n = 111) were receiving disability support. The socioeconomic status of the majority of participants is low with 78.5% (n = 223) having a mean monthly household income of <$2000. With respect to diabetes treatment, 91.4% (n = 265) of participants were being treated for type 2 diabetes and 8.6% (n = 25) were being treated for type 1 diabetes. The average body mass index (BMI) in this sample was 34.5 (SD = 8.6).

**Table 1:** Demographic, psychiatric and legal characteristics of 290 African‐American females surveyed during 2013‐2015 in the waiting room of a diabetes clinic in Atlanta

	Frequency	% (N)
Age (N = 290)		
18‐29	10	3.4
30‐49	88	30.3
50‐64	186	64.1
65	6	2.1
Education (N = 289)		
<12th	45	15.6
12th or high school grad	103	35.6
GED	15	5.2
Some college or tech school	77	26.6
Tech school grad	17	5.9
College grad	21	7.3
Grad school	11	3.8
Employment (N = 289)		
No	212	73.4
Yes	77	26.6
Current disability support (N = 289)		
No	178	61.6
Yes	111	38.4
Monthly income (N = 284)		
$0‐249	37	13.0
$250‐499	19	6.7
$500‐999	82	28.9
$1000‐1999	85	29.9
$2000 or more	61	21.5
Psychiatric hospitalization (N = 289)		
No	250	86.5
Yes	39	13.5
Suicide attempt (N = 289)		
No	248	85.8
Yes	41	14.2
Ever been arrested (N = 288)		
No	175	60.8
Yes	113	39.2

We also examined self‐reported history of psychiatric hospitalization and attempted suicide as basic indicators of psychiatric morbidity (Table 1). In our sample, 13.5% (n = 39) of participants had been hospitalized for psychiatric reasons and 14.2% (n = 41) had attempted suicide. Arrests are common in the sample, with 39.2% (n = 113) of participants reporting at least one arrest in their lifetime.

### 3.2 Prevalence of childhood and nonchildhood abuse trauma exposure

Table 2 summarizes the lifetime prevalence and rates of various trauma exposures as measured with the TEI and CTQ. With respect to nonchildhood abuse trauma exposure, 51.4% (n = 146) of our sample reported experiencing a serious accident or injury, which was the most frequently reported form of trauma exposure. Experiencing a sudden life‐threatening illness was the second most often type of trauma exposure reported by our sample (n = 115). Our assessment of childhood trauma exposure with the CTQ indicated that 23.8% (n = 67) of participants experienced at least one incident of either sexual or physical abuse and that 12.5% (n = 35) of participants had experienced both physical and sexual abuse.

**Table 2:** Nonchildhood abuse and childhood trauma exposure for current sample as assessed by the Traumatic Events Inventory (TEI)

	Frequency	% (N)
Serious accident or injury (N = 284)	146	51.4
Natural disaster (N = 284)	78	27.5
Sudden life‐threatening illness (N = 289)	115	40.6
Military combat in a war zone (N = 280)	1	0.4
Close friend or family member murdered (N = 280)	27	9.3
Attacked with a weapon by romantic partner (N = 280)	44	15.2
Attacked with a weapon by a stranger (N = 280)	55	19.0
Witnessed family member attacked with a weapon (N = 279)	42	15.1
Attacked by a romantic partner without weapon (N = 280)	97	34.6
Beaten as a child (N = 276)	46	16.7
Witnessed violence between parents (N = 277)	100	36.1
Sexual contact before 13 (N = 273)	80	29.3
Forced sexual contact between 14‐17 (N = 274)	66	24.1
Forced sexual contact after 17 (N = 275)	51	17.6

### 3.3 Associations between trauma exposure and post‐traumatic stress and depressive symptoms

We found that 30.4% (n* = *80) and 29.3% (n* = *76) of the overall sample had current PTSD and MDD, respectively, based on categorical diagnoses from PSS and BDI. Furthermore, exposure to increasing types of childhood and nonchildhood abuse trauma significantly impacted PTSD and depression symptoms in adulthood (Table 3). More specifically, exposure to increasing types of childhood trauma was associated with significantly increased adult PTSD (*P* < .001; Table 3) and MDD symptoms (*P* < .001; Table 3). Increasing exposure to different types of nonchildhood abuse trauma exposure was only associated with greater PTSD symptoms (*P* = .003; Table 3) and not MDD symptoms (*P* = .11; Table 3).

**Table 3:** Association between childhood and nonchildhood abuse trauma exposure and PTSD and depression symptoms. Letters denote significantly different values at *P* < .05

	PTSD symptoms		Depression symptoms	
	Mean ± SEM	*P*‐value	Mean ± SEM	*P*‐value
Childhood trauma				
0 types	9.69 ± 0.79^a^	<.001	11.1 ± 0.77^a^	<.001
1 type	12.4 ± 1.35^b^		14.7 ± 1.60^b^	
≥2 types	23.3 ± 0.72^c^		24.8 ± 1.97^c^	
Nonchildhood abuse trauma				
0 types	7.33 ± 3.32^a^	.003	7.71 ± 2.33	.11
1 type	6.46 ± 1.45^a^		11.4 ± 2.11	
≥2 types	13.6 ± 0.80^b^		14.8 ± 0.81	

Note

Trauma type refers to the number of different categories of abuse (physical, sexual, emotional) the individual experienced.

### 3.4 Associations between PTSD and MDD and haemoglobin A1c

**Figure 1:**
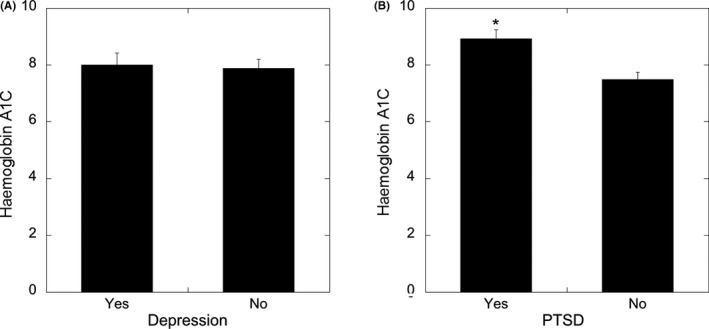
Association between MDD (A) and PTSD (B) and haemoglobin A1C concentrations relevant to T2DM in subsample of 96 women. *P ≤ .05; **P ≤ .01; ***P ≤ .001

In the subgroup of women with type 2 diabetes that underwent a structured clinical interview (n = 96), we found that 35.1% (n = 33) had current PTSD and 25% (n* = *23) had current MDD. As shown in Figure 1, current diagnosis of MDD was not associated with elevated haemoglobin A1c (*P* = .62; Figure 1A). In contrast, current diagnosis of PTSD was associated with elevated haemoglobin A1c (*P* = .002; Figure 1B). Haemoglobin A1C was significantly, but weakly, associated with history of psychiatric hospitalization (r* =* −.141, *P* = .027) and was not associated with any other trauma variables or socioeconomic variables (*P's* > .05). Furthermore, A1C was not significantly correlated with BMI (r* = *−.51*, P* = .426).

## 4 DISCUSSION

The current study examined the rates of trauma exposure, PTSD and MDD in an urban, African American and impoverished, diabetic population. In this study, we found that diabetic African‐American women with low socioeconomic status have experienced high levels of childhood and nonchildhood abuse trauma, including exposure to a serious accident or injury (51.4%), a sudden life‐threatening illness (40.6%) and sexual contact under the age of 13 (29.3%). These high rates of childhood and nonchildhood abuse trauma exposure were associated with increased PTSD and depression symptoms in the overall sample. Importantly, PTSD in type 2 diabetes women was associated with higher haemoglobin A1c. Overall, these data suggest that exposure to trauma and trauma‐related psychopathology may be key factors that influence diabetes control and risk of long‐term complications in women with type 2 diabetes.

Previous studies of trauma‐exposed populations have found an association between psychopathology and type 2 diabetes. For example, in a study of asylum seekers in the Netherlands, PTSD was more likely to be diagnosed in those individuals with type 2 diabetes.^28^ In a longitudinal study of US military service members, the incidence of diabetes among veterans with PTSD increased twofold over 3 years.^29^ Furthermore, metabolic syndrome can develop as a result of trauma exposure in marginalized groups, as a PTSD diagnosis increases risk for developing metabolic syndrome.^30^ Importantly, MDD is also highly comorbid with type 2 diabetes and can increase the risk for developing metabolic syndrome.^31^ Together these data suggest that underlying changes in biology due to PTSD and MDD contribute to individual risk for developing or exacerbating type 2 diabetes.

The high comorbidity between PTSD and MDD with metabolic disease suggests that underlying changes in biology that are common to both conditions, such as dysregulation of the hypothalamic‐pituitary‐adrenal (HPA) axis and increased inflammation, may explain the aetiology of the high comorbidity of these mental and physical disease states.^32^ Indeed, increased systemic inflammation is associated with both PTSD^33^ and MDD.^34^ Recently published findings from the substudy of women with type 2 diabetes (n = 55) described in the current manuscript show that higher concentrations of CRP are associated with PTSD and MDD.^24^ Additionally, individuals with PTSD and MDD display altered sensitivity to glucocorticoids that can lead to increases in abdominal fat and insulin resistance.^30^ Inflammation and neuroendocrine dysfunction are also risk factors for type 2 diabetes.^19^, ^35^ While this dysregulation of the HPA axis and inflammatory system has been implicated in increased risk for metabolic disorders in those with psychopathology, prospective longitudinal studies are necessary to elucidate the causal mechanisms underlying increased risk for type 2 diabetes following the development of MDD and PTSD.

In the current study, PTSD was associated with increased haemoglobin A1c. These data corroborate previous reports indicating that the presence of MDD and PTSD exacerbates the peripheral concentrations of biological factors associated with cardiometabolic disease.^36^ Our result that PTSD was associated with increased haemoglobin A1c substantiates previous findings reporting worsened glycaemic control in individuals with PTSD.^37^, ^38^ These differences in biological factors linked to metabolic disease due to MDD and PTSD may be due to alterations in HPA and inflammatory system function as outlined above. Indeed, acute stress exposure increases the release of pro‐inflammatory cytokines (TNF‐α, IL‐6 and IL‐1β)^39^ that may then worsen hyperglycaemia by increasing insulin resistance.^40^ Future studies are necessary to disentangle the mechanism by which MDD and PTSD can exacerbate haemoglobin A1c in women with type 2 diabetes.

The finding that PTSD is associated with elevated haemoglobin A1c in women with type 2 diabetes may also be due to increased medication nonadherence, a factor that was not assessed in the current study. MDD and PTSD are both associated with increased psychiatric medication nonadherence even after adjusting for sociodemographic factors, social support, alcohol use and medical comorbidities.^41^ Adherence to diabetes medication is also adversely impacted by the presence of MDD.^42^ Thus, while our results are limited by the lack of information about adherence to both psychiatric and diabetic medications, the high exposure of trauma and rates of psychopathology in the current sample could influence medication adherence across all participants. Rates of trauma exposure and psychopathology in the current sample were comparable to those we have previously reported in the primary care clinics at Grady Memorial Hospital.^4^ However, rates of PTSD vary widely by group, with the average ranging from 7.8% to 14% for women in the Detroit Area Trauma Study.^5^

A further intriguing question, unable to be directly posed in the present study, is the possibility that medications used for the treatment of type 2 diabetes may impact the risk for, or severity of, PTSD. Angiotensin‐converting enzyme (ACE) inhibitors as well as angiotensin receptor blockers (ARBs) have well‐validated utility in preventing or slowing the progression of diabetes‐related kidney disease.^43^ Interestingly, prescription of either ACE inhibitors or ARBs, but not other antihypertensive medications, has been associated with reduced PTSD symptom severity when controlling for level of prior childhood and adult trauma exposure and demographic variables in participants recruited from a heavily trauma‐exposed primary care population.^44^ Recently reported data^45^ from a separate large cross‐sectional study of trauma‐exposed civilians have demonstrated the presence of enduring alterations of the renin‐angiotensin‐aldosterone system (RAAS) in adults exposed to trauma during childhood and/or adulthood providing an empirical foundation for the association of ACE inhibitor and ARB prescription with reduced PTSD symptom severity. Further, translational research investigating the impact of acute administration of the ARB, losartan, on the acquisition^46^ and extinction^47^ of fear in healthy human participants has provided support for a possible role of ARBs in the prevention and treatment of PTSD by way of effects on the RAAS.

In summary, rates of trauma exposure and psychopathology are high in diabetic, primarily African‐American individuals of low SES and can adversely impact regulation of blood glucose. However, there are additional limitations of this study that influence the interpretation and impact of the current findings. First, the sample homogeneity limits the generalizability of the current findings to African‐American women of low SES receiving care in an inner city clinic. However, this homogeneity is also a strength of the current study, as this population is more at risk for adverse mental and physical health outcomes,^4^ and women are more susceptible to developing PTSD than men.^48^, ^49^ Our results highlight the importance of understanding the consequences of trauma exposure in a specific population with limited access to health care. Second, because the sample is comprised of female participants, it would have been ideal to include analyses examining whether there are effects of menstrual status on the outcome variables of interest. Finally, we used a cross‐sectional approach to retrospectively examine the prevalence of trauma exposure, PTSD and MDD in participants recruited from the diabetes clinic. This cross‐sectional study design does not allow us to address causality—it neither reveals whether trauma exposure and psychopathology increase an individual's risk for type 2 diabetes, nor does it address how type 2 diabetes affects depression or PTSD symptoms. However, the current findings highlight the importance of further understanding the mechanism by which trauma exposure and psychopathology exacerbate biological risk factors of diabetes. Identifying these mechanisms could have significant implications for future treatment of type 2 diabetes in those with psychopathology,^32^ as recent studies suggest that alleviation of depression symptoms via treatment can have beneficial effects on cardiometabolic factors^50^ and that significant reductions in PTSD symptoms (upon treatment for PTSD or spontaneous improvement) are associated with decreased long‐term risk for developing type 2 diabetes.^51^

## Data Availability

The data that support the findings of this study are available from the corresponding author upon reasonable request.
